# Prognosis of patients with prolonged disorders of consciousness after brain injury: a longitudinal cohort study

**DOI:** 10.3389/fpubh.2024.1421779

**Published:** 2024-07-24

**Authors:** Dong Yan, Liu Simei, Bai Hongzhao, Du Hongyan, Ding Yuchao

**Affiliations:** Department of Rehabilitation Medicine, Hospital of Zhejiang People’s Armed Police, Hangzhou, China

**Keywords:** brain injury, prolonged disorders of consciousness, prognosis, mortality, disability

## Abstract

**Background:**

The findings regarding the prognosis of prolonged disorders of consciousness (PDOC) vary widely among different studies. This study aims to investigate the mortality, consciousness recovery and disabilities of patients with PDOC after brain injury.

**Methods:**

A total of 204 patients with PDOC were included in a longitudinal cohort study, including 129 males and 75 females. There were 112 cases of traumatic brain injury (TBI), 62 cases of cerebral hemorrhage (CH), 13 cases of cerebral infarction (CI) and 17 cases of ischemic hypoxic encephalopathy (IHE). The status of consciousness at 1, 2, 3, 6, 12, 18, 24, 36, 48 months of the disease course was assessed or followed up using the Revised Coma Recovery Scale (CRS-R). If the patients were conscious, the disability Rating Scale (DRS) was also performed. The prognosis of different PDOC including coma, vegetative state (VS) and minimal conscious state (MCS) was analyzed. The survival patients were screened for variables and included in multivariate binary Logistic regression to screen the factors affecting the recovery of consciousness.

**Results:**

The mortality rates at 12, 24, 36, and 48 months were 10.7, 23.4, 38.9, and 68.4%, respectively. The median time of death was 18 months (8.75, 29). The probability of MCS regaining consciousness was higher than VS (*p* < 0.05), with the degree of disability left lower than VS (*p* < 0.05). There was no significant difference between MCS− and MCS+ groups in terms of the probability of regaining consciousness, the extent of residual disability, and mortality rates (*p* > 0.05). The mortality rate of coma was higher than that of other PDOC (*p* < 0.05). The mortality rate of MCS was lower than that of VS, but the difference was not statistically significant (*p* > 0.05). The probability of consciousness recovery after TBI was the highest and the mortality rate was the lowest. The possibility of consciousness recovery in IHE was the least, and the mortality rate of CI was the highest. The cause of brain injury and initial CRS-R score were the factors affecting the consciousness recovery of patients (*p* < 0.05).

**Conclusion:**

The prognosis of MCS is more favorable than VS, with comparable outcomes between MCS− and MCS+, while comatose patients was the poorest. TBI has the best prognosis and IHE has the worst prognosis.

## Introduction

Acute disorders of consciousness (DOC) are often caused by cerebral pathology, such as traumatic brain injury (TBI), ischemic hypoxic encephalopathy (IHE) and stroke. Due to the advancement in medical care in China, an increasing number of patients with DOC have survived. Prolonged DOC (PDOC) is defined as consciousness disorder persisting for more than 28 days following severe craniocerebral injury (1).

PDOC encompasses coma, vegetative state (VS)/unresponsive wakefulness syndrome (UWS), and minimally conscious state (MCS) (2). Coma is defined as the total absence of arousal and awareness, characterized by the absence of eye opening (3). VS/UWS is characterized by wakefulness without awareness, exhibiting no voluntary or purposeful behavioral responses to visual, auditory, or tactile stimuli (4). Patients in MCS exhibit non-reflexive or purposeful behaviors, yet they are unable to communicate effectively (5). MCS is further categorized into two sub-states: MCS+ (characterized by high-level behavioral responses, such as command following) and MCS− (exhibiting low-level behavioral responses, including visual pursuit and pain localization) (6). Emergence from MCS (EMCS) occurs when the patient demonstrates reliable communication or the functional use of two objects (7).

Patients with PDOC can survive for prolonged duration. Given the significant rise in the global prevalence of these patients, investigations into the progression of PDOC and the quest for prognostic methods are particularly pertinent. The findings regarding the prognosis of PDOC vary widely among different studies. A German study involving 102 patients with PDOC who were followed for 2–14 years post-discharge reported that 71% of patients either decreased or exhibited no improvement, while 29% regained consciousness, including six patients who regained consciousness 3 years after onset. Furthermore, the study found that patients with MCS tended to improve more frequently and to a better outcome compared to those with VS/UWS (8). A South Korean study including 50 patients with brain injury revealed that 46% of the participants emerged from PDOC within 200 median days following injury onset, and no patient succumbed during that period. The prognosis for patients with MCS was more favorable than for those with VS/UWS. However, patients who were diagnosed with coma upon admission and exhibited neurological or medical instability were excluded from this study (9). Researchers in Russia conducted a one-year follow-up study involving 211 patients with PDOC and discovered that mortality reached 35% within 1 year of the event. The primary predictors of survival were younger age and the initial level of consciousness. No significant correlations were observed between survival, enhanced consciousness, and gender (10). A French study involving 67 patients with DOC within 90 days revealed that both survival rates and outcomes were notably superior in the MCS group compared to the VS/UWS group. Better prognoses were associated with clinical status and age, rather than etiology (11). A Chinese study encompassing 93 patients who were followed for 12–37 months reported that 33 patients regained full consciousness, while 7 succumbed within 12 months of injury. The potential for unfavorable outcomes was significantly higher in patients with VS/UWS compared to those with MCS (12). Notably, limited research has investigated the prevalence of disability post-consciousness recovery among patients experiencing PDOC.

To investigate the recovery of consciousness, the degree of disability, and to identify the prognosis of different states of consciousness, we conducted this long-term follow-up of patients with PDOC.

## Materials and methods

This study has the following inclusion criteria: (1) Patients with DOC resulting from brain injury for a duration of over 1 month. (2) age ≥ 18 years. (3) Patient whose level of consciousness should be evaluated face-to-face at the first and second months following the onset of the condition. (4) Signed informed consent from the patients’ families indicating agreement to participate in the study.

The exclusion criteria are as follows: (1) Patients who regain consciousness within 1 month after the onset of the condition (2) Patients combined with spinal cord injury (3) Patients whose state of consciousness cannot be evaluated face-to-face at the first and second months of the disease course (4) Follow-up duration of less than 6 months.

A total of 204 patients admitted to the rehabilitation department of the Hospital of Zhejiang People’s Armed Police, Hangzhou, China were enrolled in this study, spanning from September 2019 to August 2023. The follow-up period concluded in February 2024.

### Data collection

The status of consciousness was evaluated utilizing the revised Coma Recovery Scale (CRS-R), with scores indicating VS, MCS−, MCS+, or EMCS. If the patient fails to open their eyes in response to any stimulation and lacks other signs of MCS, the coma status is documented. Patients exhibiting EMCS were further evaluated using the Disability Rating Scale (DRS).

All subjects were enrolled in the study at the first month of their disease course. Face-to-face assessments were conducted at enrollment, 1 month later (when the disease course was 2 months), and subsequently at 3, 6, 12, 18, 24, 36, and 48 months of the disease course. Face-to-face assessments were conducted for patients who remained hospitalized, while telephone follow-ups were arranged for those who were discharged. The evaluation and follow-up period extended until February 2024. If a patient deceased, the duration of their disease (in months) at the time of death was recorded. If the precise time of death could not be determined during follow-up, the follow-up time was considered as the time of death.

Follow-up was conducted by skilled neurologists. During the follow-up, it is recommended that family members utilize the We-chat mini program developed in the early stages to obtain information on the patients’ state of consciousness (13). The neurologists will meticulously inquire into key assessments, including visual pursuit, localization to noxious stimulation, autonomic motor response, reproducible movement upon command, functional object use, and precise communication. The result will be compared with the previous assessment to determine whether there is progress. The neurologists will ultimately determine the patient’s state of consciousness based on the aforementioned information. If the patient regains consciousness, the DRS assessment shall be continued.

### Statistical analysis

IBM SPSS software, version 23 (IBM, New York), was utilized for statistical analysis. Normally distributed quantitative data were presented as the mean ± standard deviation. Quantitative data that did not follow a normal distribution were described by the median (first quartile, third quartile). Categorical data (sex, number of cases) were expressed as frequencies (%). The non-parametric Kruskal-Wallis test was employed for variables that did not follow a normal distribution. Fisher exact test was used for categorical variables. The Monte Carlo test was utilized when the Fisher test was not computable, and the Bonferroni test was employed for pairwise comparisons among multiple data groups. Multiple categorical variables were transformed into dummy variables and included in the Logistic regression analysis. The Hosmer-Lemeshow fit and goodness-of-fit test was employed to assess the regression equation. All reported *p* values were two-tailed with a significance threshold of 0.05.

## Results

### Demographical and clinical data

The demographic and clinical data of the patients participating in the study are summarized in Table 1. The follow-up time, number of cases, and mortality are presented in Table 2. The mortality rates at 12, 24, 36, and 48 months were 10.7, 23.4, 38.9, and 68.4%, respectively. The median time of death was 18 months (8.75, 29).

**Table 1:** Demographical and clinical data of patients.

Median age	60.5 (50, 68)				
Sex (male/female)	129/75				
Etiology	Total (male/female)	Coma	VS/UWS	MCS−	MCS+
Traumatic brain injury	112 (68/44)	13	49	46	4
Cerebral hemorrhage	62 (39/23)	7	26	26	3
Cerebral infarction	13 (11/2)	3	4	6	0
Ischemic hypoxic encephalopathy	17 (11/6)	0	14	2	1
Total level of consciousness	204	23	93	80	8
Median CRS-R score		1 (0, 2)	4 (4, 5)	10 (8, 12)	16 (13.5, 16.25)

**Table 2:** Follow-up time and mortality.

Follow-up time	12 months	24 months	36 months	48 months
Cases	187	156	121	76
Deaths	20	37	47	52
Mortality	10.7%	23.4%	38.9%	68.4%

### Relationship between state of consciousness at 1 month of disease course and prognosis

Due to the extensive follow-up time, patients may have emerging diseases during follow-up that could impact their level of consciousness. Therefore, the optimal state of consciousness during the recovery process is utilized as prognostic data for patients in the data analysis.

The differences in the prognosis among the four conscious states were statistically significant (*F* = 35.479, *p* < 0.05). Pairwise comparison revealed no significant difference between the coma and VS groups (*p* > 0.05) or between the MCS− and MCS+ groups (*p* > 0.05). However, a statistically significant difference was observed in the prognostic outcomes between the coma/VS and MCS−MCS+ groups (*p* < 0.05). The probability of consciousness recovery was higher in the MCS−/MCS+ group compared to the coma/VS group (Table 3).

**Table 3:** State of consciousness at 1 month of disease course and prognosis.

State of consciousness at 1 month of disease course	Optimal consciousness (N)		Total	Consciousness recovery ratio
	Not EMCS	EMCS		
Coma	10^a^	0^a^	10	0%
VS/UWS	46^a^	20^a^	66	30.3%
MCS−	21^b^	48^b^	69	69.6%
MCS+	1^b^	6^b^	7	85.7%

*p* < 0.05 when a compared to b.

VS, Vegetative State; UWS, Unresponsive Wakefulness Syndrome; MCS, Minimally Conscious State.

### Relationship between state of consciousness at 2 months of disease course and prognosis

As the duration of consciousness disturbance increases, the likelihood of regaining consciousness decreases, as evident in Table 4. Significant differences were observed in the prognosis among the four conscious states (*F* = 27.280, *p* < 0.05). Pairwise comparison showed no significant differences between the coma and VS groups (*p* > 0.05), coma and MCS− groups (*p* > 0.05), or between the MCS− and MCS+ groups (*p* > 0.05). However, significant differences were observed in the prognosis between the coma and MCS+, VS and MCS−, and VS and MCS+ groups (*p* < 0.05).

**Table 4:** State of consciousness at 2 months of disease course and prognosis.

State of consciousness at 2 months of disease course	Consciousness (N)		Total	Consciousness recovery ratio
	Not EMCS	EMCS		
Coma	5^a,b^	0^a,b^	5	0%
VS/UWS	46^a^	7^a^	53	13.2%
MCS−	26^b,c^	26^b,c^	52	50%
MCS+	4^c^	10^c^	14	71.4%

*p* < 0.05 among a, b, and c.

VS, Vegetative State; UWS, Unresponsive Wakefulness Syndrome; MCS, Minimally Conscious State.

The ratio of consciousness regain indicates that a higher proportion of patients with MCS−/MCS+ regained consciousness compared to those in coma/VS, echoing the findings of the first month.

Contrary to the initial month’s findings, the prognosis of coma patients differed significantly from those with MCS− during the first month, but not in the second month.

### Relationship between the state of consciousness at the first month and later disability in patients who regain consciousness

Seventy-four patients with disturbed consciousness in the first month recovered to EMCS during follow-up. Among them, 20 patients with VS had a median DRS score of 13.5 (range: 8.75, 18.25), 48 patients with MCS− had a DRS score of 8 (range: 4.75, 13.25), and six patients with MCS+ had a median DRS score of 4.5 (range: 3, 12.75). Significant differences were observed in DRS scores among the three groups (*p* < 0.05). Specifically, there was no significant difference between the MCS− and MCS+ groups (*p* > 0.05), whereas significant differences were observed between the MCS− and VS groups, as well as between the MCS+ and VS groups (*p* < 0.05).

The findings indicate that a better initial state of consciousness is associated with lower levels of disability. Specifically, patients with VS exhibit higher levels of disability compared to those with MCS upon regaining consciousness. However, there is no significant difference in disability levels between patients with MCS− and MCS+.

### Comparison of initial consciousness disturbance between survival group and death group

The composition of initial consciousness disturbance differed significantly between the survival and death groups (*F* = 17.949, *p* < 0.05). Pairwise comparisons revealed significant differences between coma and MCS− as well as coma and MCS+ (*p* < 0.05). No significant differences were observed between VS and MCS− or VS and MCS+ (*p* > 0.05) (Table 5). The highest mortality rate was observed among coma patients (56.5%), while the lowest mortality rate was found among MCS+ patients (0%). A positive correlation was observed between the initial state of consciousness and mortality, with better consciousness states associated with lower mortality rates.

**Table 5:** Comparison of initial consciousness disturbance between survival and death group.

State of consciousness at 1 month of disease course	Survivals	Deaths	Total	Mortality
Coma	10^a^	13^a^	23	56.5%
VS/UWS	66^a,b^	27^a,b^	93	29.0%
MCS−	69^b^	12^b^	81	14.8%
MCS+	7^b^	0^b^	7	0%
Total	152	52	204	25.5%

*p* < 0.05 between a and b.

VS, Vegetative State; UWS, Unresponsive Wakefulness Syndrome; MCS, Minimally conscious state.

### Comparison of the conscious state at second month between the survival group and the death group

Significant differences in mortality were observed among patients with varying states of consciousness at the second month (*F* = 28.153, *p* < 0.05). Pairwise comparisons demonstrated significant differences between coma and other consciousness disorders (*p* < 0.05), as well as between VS and EMCS (*p* < 0.05). No significant differences in mortality were observed between MCS (MCS− and MCS+) and VS patients (*p* > 0.05) (Table 6).

**Table 6:** Comparison of consciousness state at second month between survival and death group.

State of consciousness at second month of disease course	Survivals	Deaths	Total	Mortality
Coma	5^a^	12^a^	17	70.6%
VS/UWS	54^b^	25^b^	79	31.6%
MCS−	52^b,c^	11^b,c^	63	17.5%
MCS+	13^b,c^	3^b,c^	16	18.7%
EMCS	28^c^	1^c^	29	3.5%
Total	152	52	204	25.5%

*p* < 0.05 between a, b, and c.

VS, Vegetative State; UWS, Unresponsive Wakefulness Syndrome; MCS, Minimally conscious state; EMCS, Emergence from minimally conscious state.

Patients who remained in a coma at the second month exhibited a further increase in mortality risk, reaching up to 70.6%. Even patients who regained consciousness during this period were still at risk of death.

### Relationship between etiology and prognosis of brain injury

Significant differences were observed in the prognoses of patients with different etiologies of brain injury (*F* = 24.808, *p* < 0.05). Among these patients, those with TBI exhibited the highest probability of consciousness recovery and the lowest mortality rate. Patients with IHE exhibited the lowest likelihood of consciousness recovery. Patients with cerebral infarction (CI) exhibited the highest mortality rate compared to other etiologies. See Table 7 for detailed mortality rates across different etiologies.

**Table 7:** Etiology and prognosis of brain injury.

Etiology	Total	VS/WUS	MCS−	MCS+	EMCS	Recovery of consciousness	Mortality
Cerebral hemorrhage	62	6	14	4	23	37.1%	24.2%
Traumatic brain injury	112	16	19	5	48	42.9%	21.4%
Cerebral infarction	13	0	4	0	2	15.4%	53.8%
Ischemic hypoxic encephalopathy	17	7	2	1	1	5.9%	35.3%
Total	204	29	39	10	74	36.3%	25.5%

VS, Vegetative State; UWS, Unresponsive Wakefulness Syndrome; MCS, Minimally conscious state; EMCS, Emergence from minimally conscious state.

### Factors influencing recovery of consciousness

To explore the relationships between baseline predictors and outcomes among surviving patients (*n* = 152), a regression model was employed. Selected variables such as gender, age, etiology, and initial CRS-R scores were evaluated as independent predictors of consciousness recovery. Univariate regression analysis was initially conducted, with the findings presented in Table 8. These results indicated that age and gender were not significantly associated with regaining consciousness, whereas etiology and initial CRS-R scores could potentially influence the outcome. Dummy variables were assigned as follows: etiology (TBI = 1, cerebral hemorrhage (CH) = 2, CI = 3, IHE = 4); and outcome (recovery of consciousness = 1, non-recovery of consciousness = 2). Multivariate regression analysis revealed that etiology and higher initial CRS-R total scores were significantly associated with consciousness recovery. These results are presented in Table 9. The logistic regression model was formulated as Logit (p) = 0.816 + 0.68 × etiology - 0.261 × initial CRS-R score. Hosmer-Lemeshow fit and goodness test indicated that the regression prediction model had a good fit (*F* = 6.659, *p* > 0.05).

**Table 8:** Single factor comparison between consciousness recovery group and non-recovery group.

	Sex		Median age	Etiology				CRS-R
	M	F		TBI	CH	CI	ICH	
Recovery	45	29	57.5 (48.75, 66.75)	48	23	2	1	9 (6, 12.75)
Non-recovery	45	33	62 (50, 69)	40	24	4	10	5 (3, 7)
*X*^2^/*Z*	0.153		−1.518	8.914				−5.2
*p* value	0.696		0.129	0.026				0.000

M, Male; F, Female; TBI, Traumatic brain injury; CH, Cerebral Hemorrhage; CI, Cerebral infarction; IHE, ischemic hypoxic encephalopathy; CRS-R, Revised Coma Recovery Scale.

**Table 9:** Multi-factor analysis of consciousness recovery.

Factor	B (partial regression coefficient)	standard error (S.E.)	Wald *X*^2^	*p*	OR (95%CI)
Etiology	0.680	0.249	7.421	0.006	1.973 (1.210, 3.218)
Initial CRS-R score	−0.261	0.053	24.477	0.000	0.770 (0.694, 0.854)

## Discussion

This study investigated the long-term survival, recovery of consciousness, and degree of disability among patients with PDOC. A comparison was made between the prognoses of different chronic consciousness states. The study found that the likelihood of regaining consciousness was higher among patients with MCS compared to those with VS/UWS. Furthermore, patients with MCS exhibited a lower degree of disability compared to those with VS/UWS. Overall, the prognosis for patients with MCS was more favorable. Although the mortality rate among patients with MCS was lower than that of VS/UWS, this difference was not statistically significant. MCS− and MCS+ groups exhibit similar outcomes in the aforementioned aspects.

Acute DOC often exhibit a higher potential for recovery. Among individuals who exhibited VS/UWS at the 2-week mark, a significant 78% (62 of 79) regained consciousness within 12 months (14). Another comprehensive study reported that 82% of patients with moderate to severe TBI regained consciousness by the conclusion of inpatient rehabilitation (15). In contrast, our study revealed the probability of regaining consciousness after the initial month of the illness among patients exhibiting VS/UWS was approximately 30.3%, and this figure decreased to 13.2% in patients still in VS/UWS at 2 months. These percentages are notably lower than the 78% reported in the acute phase studies. Furthermore, the likelihood of regaining consciousness with MCS− and MCS+ at 1 month was 69.6 and 85.7%, respectively, and these probabilities decreased to 50 and 71.4% in patients still in MCS− or MCS+ at 2 months. These findings suggest that the earlier the patient exhibits a better state of consciousness, the higher the chances of regaining consciousness in the later stages.

This study revealed that 13.2% of patients with VS/UWS regained consciousness by the second month, while some experienced prolonged unconsciousness lasting over a year. Kudre’s research demonstrates that delayed improvements in awareness are not uncommon among non-traumatic cases of PDOC (16). This justifies the need to replace the term “permanent VS” with “chronic VS/UWS,” specifying the duration, as recommended in the guideline (1).

The study by Pan et al. demonstrates that patients with cognitive motor dissociation identified through brain-computer interface have a significantly better prognosis, with a higher rate of recovery of consciousness compared to non-cognitive motor dissociation patients (17). The research indicates that brain-computer interface could serve as an effective prognostic indicator for consciousness recovery in patients with DOC, potentially aiding in family counseling, decision-making, and rehabilitation program design (18). Nevertheless, the operation of a brain-computer interface is intricate, and the necessary conditions are not present in the majority of institutions.

Numerous guidelines recommend the utilization of CRS-R for evaluating consciousness in patients with PDOC (1, 2, 19). However, the CRS-R does not effectively distinguish between VS/UWS and coma. Coma represents the most severe disorder of consciousness, characterized by the absence of eye opening, whereas patients in VS/UWS can open their eyes autonomously or in response to stimulus. In our study, patients who experienced painful stimuli but did not open their eyes or achieve a MCS score were separately categorized as coma, in order to differentiate them from patients in VS/UWS. The findings revealed no significant difference between coma and VS/UWS patients at 1 month; however, a higher proportion of VS/UWS patients exhibited improved consciousness compared to coma patients, while the mortality rate was lower in VS/UWS patients. Notably, if a patient remains in coma by the second month, the subsequent mortality rate is significantly higher compared to patients in VS/UWS. Consequently, it is recommended that patients with PDOC should be accurately distinguished between coma and VS/UWS.

The prognosis of PDOC is influenced by numerous factors, the consistency of which varies across studies. Numerous studies have suggested a correlation between age and prognosis, with youth being a factor associated with improved consciousness (11, 12, 20–26). Additional prognostic factors encompass gender (20), a brief disease duration (20, 22), and a high CRS-R score (12, 22, 23, 25, 26). The association between the cause of brain injury and prognosis remains controversial. Some studies argue that the cause of brain injury is unrelated to prognosis (21, 22), whereas others maintain that it is a crucial determinant of prognosis (26, 27). The Feng Zhen team developed a predictive model that may assist in forecasting 3-year outcomes for patients with PDOC, incorporating independent prognostic factors such as age, Glasgow coma scale score, level of consciousness, and brainstem auditory-evoked potential grade (28). The research by Whyte reveals that the DRS score at enrollment and the rate of DRS score change in the early weeks post-enrollment are key predictive factors for recovery outcomes in patients with prolonged posttraumatic DOC (29). Regression analysis conducted in this study identified the primary factors influencing consciousness recovery to be the etiology of injury and the patient’s initial level of consciousness. However, age and gender were found to be unrelated to consciousness recovery. A positive correlation was observed between the initial state of consciousness and the likelihood of consciousness recovery, with a better initial state associated with a higher chance of recovery and a lower severity of disability. Patients with TBI exhibited the highest potential for consciousness recovery, whereas patients with IHE demonstrated the lowest potential for consciousness recovery.

Of the 74 patients who regained consciousness, only 1 case (1.3%) achieved a DRS score of 0, indicating full social participation, while 10 cases (13.5%) were able to take care of themselves but were unable to regain their work ability. One possible explanation for this may be that some patients are older and do not have the obligation to support the family, thus eliminating the need to participate in work. Despite the fact that the majority of patients with PDOC (85.1%) regained consciousness, significant dysfunction persisted, affecting their ability to live independently and participate socially. Therefore, it is crucial to focus on addressing the remaining disabilities among patients with PDOC following the restoration of consciousness.

Reports of mortality rates among patients with PDOC vary significantly. A follow-up study of 143 patients with brain injury, including 55 with TBI and 88 with non-TBI (NTBI), revealed that 41 patients (28.7%) died within 24 months post-injury. The mortality rate was higher in patients with VS/UWS (42.6%) compared to those with MCS (16%) (30). A retrospective study examined 154 patients with TBI and 52 patients with non-TBI who remained in VS/UWS for over 1 month. At the end of the follow-up period, the mortality rate was 28% among patients with TBI in VS/UWS and 47% among patients with non-TBI in VS/UWS (24/51 patients). Age, duration of stay in intensive care, consciousness rehabilitation, and the presence of hydrocephalus were found to significantly affect survival rates (24). A study examining 211 patients with severe acquired brain injury reported a mortality rate of 35% within 1 year post-injury, with 20.3% of patients dying within the first 3 months. Among the patients studied, the majority (86.8%) were in VS/UWS (10). These disparities in mortality rates are likely due to variations in enrollment criteria among the different studies. Our study revealed a yearly increase in the death rate associated with PDOC, rising from 10.7% in the first year to 23.4% in the second year, and ultimately reaching 68.4% in the fourth year. The median survival time was 18 months, and a significant correlation was observed between the death rate and the severity of unconsciousness. Patients in a coma exhibited the highest mortality rate, with an increased risk of death correlating with prolonged coma duration. This high mortality rate may be attributed to severe brain injury in coma patients, which significantly impacts respiration and circulation, leading to severe systemic complications. Although MCS patients exhibited a higher likelihood of survival compared to VS/UWS patients, the difference was not statistically significant. In terms of etiology, the death rate associated with TBI was the lowest, whereas the death rate associated with CI was the highest. A plausible explanation for this finding is that CI leading to DOC often signifies severe infarction, often involving the brainstem and affecting respiration, thereby contributing to the high mortality rate.

The salient features of this study are as follows: Firstly, the prognoses of coma and VS are inconsistent, necessitating differentiation when assessing levels of consciousness. Secondly, this study reports on the residual disability conditions in patients with DOC following their recovery of consciousness. The prognosis is not favorable.

This study also has several limitations that need to be acknowledged. Firstly, consciousness was only assessed using behavioral scales, excluding instrumental techniques such as EEG, PET, and fMRI. This approach may have led to misjudgments in patients with cognitive motor dissociation. Secondly, due to the taboo nature of discussing death in Chinese culture, telephone follow-up was sometimes unable to accurately ascertain the exact date of death. Instead, follow-up time was used as a proxy, which may have introduced some inaccuracies. Additionally, determining the exact cause of death was challenging, as it could be due to complications arising from the primary intracranial injury, including infections, thromboembolism, or new diseases like CI, CH, myocardial infarction, or others. Thirdly, another limitation is the inconsistency in the endpoints of follow-up, which could potentially affect the reliability and validity of data analysis. Lastly, it is worth noting that this study was conducted at a single center, potentially limiting the generalizability of its findings to a broader population.

## Conclusion

The probability of regaining consciousness in MCS was higher than in VS/UWS, and the severity of disability after regaining consciousness was lower in MCS than in VS/UWS. Within MCS, the outcomes for both MCS− and MCS+ were comparable. Among the conditions studied, the prognosis for traumatic brain injury was the most favorable, whereas hypoxic ischemic encephalopathy had the poorest prognosis.

## Data availability statement

The raw data supporting the conclusions of this article will be made available by the authors, without undue reservation.

## Ethics statement

The studies involving humans were approved by Ethical Committee of Hospital of Zhejiang People’s Armed Police. The studies were conducted in accordance with the local legislation and institutional requirements. Written informed consent for participation in this study was provided by the participants’ legal guardians/next of kin.

## Author contributions

DoY: Conceptualization, Formal analysis, Methodology, Writing – original draft, Writing – review & editing. LS: Data curation, Investigation, Project administration, Writing – original draft. BH: Data curation, Investigation, Resources, Writing – original draft. DH: Resources, Writing – original draft. DiY: Resources, Writing – original draft.
